# Multispectral imaging for zeaxanthin content in the exocarp of chili peppers

**DOI:** 10.1016/j.fochx.2026.103992

**Published:** 2026-05-18

**Authors:** Wei Liang, Wei Gao, Xingna Lv, Jinxiang Zhao, Xuechun Tian, Shuaitao Di, Qiang Li, Dongfang Zhang, ShuangXia Luo, XuePing Chen

**Affiliations:** a Key Laboratory of Vegetable Germplasm Innovation and Utilization of Hebei, Collaborative Innovation Center of Vegetable Industry in Hebei, College of Horticulture, Hebei Agricultural University, Baoding 071000, China; b Chenguang Biotech Group CO., LTD, China

**Keywords:** Zeaxanthin (PubChem CID: 5280899), Capsanthin (PubChem CID: 5281228), Carotenoids (PubChem CID: 11227325), Multispectral imaging, Zeaxanthin, One-dimensional convolutional neural network, Multimodal feature fusion, Prediction model

## Abstract

This study developed a model to predict zeaxanthin content in peppers using multispectral imaging and chemical data. A one-dimensional convolutional neural network (1D CNN) model was identified as the optimal single-modal model after comparing four machine learning algorithms. On the prediction dataset, the model achieved a determination coefficient (*Rp*^*2*^) of 0.7639. Building upon the 1D CNN framework, a multimodal feature fusion model (MCSF) was constructed by integrating the chemical measurements of capsanthin and total carotenoid contents using a multilayer perceptron. This enhanced model demonstrated excellent predictive accuracy and robustness, with *Rp*^*2*^ values of 0.9318 and 0.9211 across different spectral ranges. For high-throughput detection purposes, a simplified model that replaced measured capsanthin with a comprehensive red index still performed well, with an *Rp*^*2*^ of 0.8912 and an *RPD* of 3.11. This strategy provides a new solution for the efficient spectral detection of plant chemicals affected by multicollinearity in their absorption spectra.

## Graphical abstract

**Figure:**
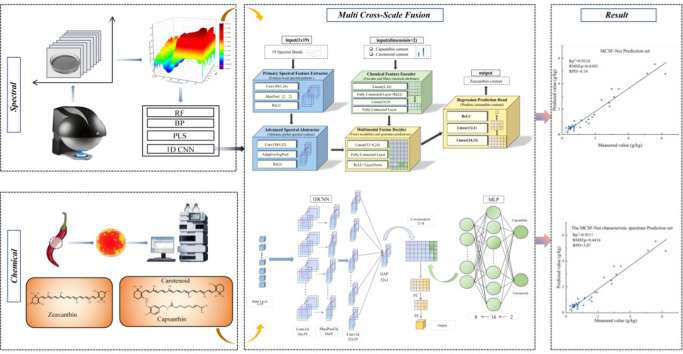

## 1 Introduction

Chili pepper (*Capsicum annuum* L.) is a cash crop cultivated worldwide. Its fruit is rich in carotenoids such as zeaxanthin and capsanthin (Ana Carolina Ana Carolina De Aguiar et al., 2022), and serves as a principal industrial raw material for zeaxanthin extraction. Zeaxanthin is a hydroxylated compound, with potent antioxidant properties which play multiple critical roles in human health (Morita et al., 2024). For example, zeaxanthin can safeguard eye health and reduce the risk of macular degeneration and cataracts (Addo et al., 2024; Hu et al., 2010; Li et al., 2013), prevent cardiovascular diseases (Li et al., 2011; Mares-Perlman et al., 2002), and improve cognitive function (Chew et al., 2015; Zhou, Huang, et al., 2017). Humans cannot synthesize zeaxanthin endogenously and must obtain it through their diet. Consequently, there is a substantial demand for zeaxanthin in the food industry. However, chili pepper varieties with high zeaxanthin content are currently scarce, and the breeding of target varieties is severely restricted by the lack of cost-effective high-throughput detection technologies.

The currently available zeaxanthin detection approaches are mainly based on high-performance liquid chromatography (HPLC) and liquid chromatography-tandem mass spectrometry (Boulet et al., 2020; Kopec et al., 2018). However, these procedures are complex, cumbersome, time-consuming, labor-intensive, costly, and environmentally harmful, making cost-efficient high-throughput sample analysis difficult to achieve. Multispectral imaging (MSI) is characterized by its simplicity, practicality, and cost-efficiency and enables the simultaneous acquisition of spatial and spectral information (Wang et al., 2021). MSI has been applied in the prediction of macronutrients in durian leaves (Phanomsophon et al., 2024), dietary fibers in Chinese cabbage (Zhang et al., 2024), and chlorophyll content in maize leaves (Tian et al., 2025) However, its application in the prediction of the zeaxanthin content of chili peppers remains unreported.

There is a close metabolic pathway relationship between zeaxanthin and capsanthin during the development of chili pepper fruit. Zeaxanthin acts as a direct precursor to capsanthin and the two share a portion of the upstream metabolic pathway (Villa-Rivera & Ochoa-Alejo, 2020). In addition, they have nearly identical chemical structures, except for their end groups, which leads to indistinguishable absorption profiles in the blue band (Jin et al., 2007; Xu et al., 2023). The absorption signal of capsanthin causes multicollinear interference, affecting the accuracy of its quantification (Zhang et al., 2022). Consequently, when detecting zeaxanthin, capsanthin must be incorporated as an auxiliary variable to correct for the spectral contribution of zeaxanthin and enhance its prediction reliability.

Multimodal feature fusion (MCSF) is a technique which integrates data from different modalities to enhance model performance (El-kenawy et al., 2024) and has been extensively applied in medical image recognition and crop yield prediction. For example, an MCSF technique based on Raman spectroscopy, Fourier transform infrared spectroscopy, and metabolomics data can enhance the diagnostic accuracy rate of diseases such as systemic lupus erythematosus (Zhou et al., 2024), thyroid neck lymph node metastasis, and cancer (Wu et al., 2025). Multimodal fusion based on spectral and visual data can increase the detection accuracy of camellia oil contaminated with rape oil (Deng et al., 2025). Multimodal data fusion based on RGB light, multispectral, and thermal sensors can improve the accuracy of soybean yield prediction (Maimaitijiang et al., 2020). However, the application of MCSF to the prediction of absorption peak multicollinear interferences has rarely been studied.

In this study, an MCSF model was created by combining spectral data with capsanthin and carotenoid content data, based on carotenoid metabolic pathway analysis and the comparative analysis of various traditional algorithms. Results showed that the MCSF model significantly enhanced the accuracy of zeaxanthin prediction. The degree of redness of the exocarp of red chili peppers is highly correlated with their capsanthin content (Lin et al., 2023). For high-throughput detection purposes, we introduced a composite red index (CRI) into this study to characterize capsanthin content in order to eliminate the multicollinear interference of capsanthin in zeaxanthin and establish a highly efficient and accurate detection model for zeaxanthin in chili peppers.

## 2 Materials and methods

### 2.1 Material collection and preparation

A total of 159 fully ripe chili peppers (*Capsicum annuum* L.) of different genotypes were included as experimental materials. Intact chili pepper fruits without disease spots or insect bite marks were harvested and rinsed with distilled water. After absorbing the excess water with paper towels, the fruits were cut open to remove the seeds and the exocarp was dried to constant weight in an oven at 55 °C. Next, the exocarp was ground to a powder in a crusher, sieved through a 50-mesh porous screen (particle size = 0.3 mm), and stored in vacuum plastic packaging at a room temperature of 25 °C. The experimental materials were provided by the Hebei Provincial Key Laboratory of Vegetable Germplasm Innovation and Utilization and planted at the Scientific Research Base of the Hebei Agricultural University in April 2024.

### 2.2 Multispectral image acquisition

The chili pepper powder was subjected to MSI using a VideometerLab instrument (VideometerA/S, Herlev, Denmark) at a wavelength range of 365–970 nm to acquire imaging data. Before imaging, the powder was evenly spread in a 9-cm petri dish to avoid any deviations in results caused by lighting and background conditions. The processing time for a single sample was 5–10 s, and the resulting spectral images had a resolution of about 41 μm/pixel and a size of 2056 × 2056 pixels. The specific operational steps involved were: 1) Warming-up the VideometerLab4 instrument for 30 min; 2) Setting the MSI working parameters; and 3) Placement of each sample in the center of the platform, with the initial descent height of the instrument's sphere and the acquisition height of the sample carrier platform set at 60 mm and 0 mm, respectively.

### 2.3 Determination of zeaxanthin and capsanthin content

The HPLC protocols followed those given in the Hebei Provincial Standard ‘Determination of trans-capsanthin content in capsanthin’ (DB13/T5159-2019) and an ultraviolet-visible spectrophotometer (UV755B, Shanghai Youke Instrument Co., Ltd., China) was used to detect the zeaxanthin, capsanthin and total carotenoid contents. In brief, samples (0.15–0.2 g) were first weighed twice, placed in a 50-mL volumetric flask, diluted to volume with HEAT solution (n-hexane, anhydrous ethanol, acetone, toluene at a ratio of 10:6:7:7 by volume), mixed by shaking, and then left in a dark place for 10 min. Thereafter, 5 mL of the resulting solution was pipetted into a new 100-mL volumetric flask and 25 mL of HEAT solution and 4 mL of 40% potassium hydroxide-methanol solution were added. The 100-mL volumetric flask was then shaken to mix the solution. Next, the 100-mL volumetric flask was placed in a thermostatic water bath at 25 °C for 30 min of saponification. Then, 30 mL of n-hexane was added, and 10% anhydrous sodium sulfate was used for dilution to volume. After vigorous shaking for 2 min, the flask was left to stand in a dark place for 1 h for stratification. Then, 5 mL of the supernatant was pipetted into a new 50-mL volumetric flask and diluted to volume with n-hexane, to create a test solution for analysis using the ultraviolet-visible spectrophotometer. The supernatant was filtered using a 0.45 μm organic filter membrane and then used as the test solution for HPLC analysis (LC-2030C3DPlus, Shimadzu, Japan). The chromatographic conditions comprised a mobile phase (prepared by mixing n-hexane, acetone, and ethyl acetate at a ratio of 74:11:15 by volume), a column temperature of 40 °C, a flow rate of 1.5 mL/min, and an injection volume of 20 μL. The measurements were repeated twice.

### 2.4 Data processing

#### 2.4.1 Spectral data extraction and preprocessing

A region in the petri dish with evenly distributed chili pepper powder was selected for spectral investigation, and its spectral wavelength data were extracted and averaged for quantitative analysis. The spectral data were preprocessed using a continuous wavelet transform, followed by Min-Max normalization. WAVE, a common method for spectral preprocessing, removes signal and background noise interference and significantly enhances data accuracy (Hegazy et al., 2016).

#### 2.4.2 Principal component analysis (PCA) for screening of characteristic spectral bands

The Kaiser-Meyer-Olkin (KMO) test and Bartlett's sphericity test were systematically performed on the spectral data to determine whether a PCA was applicable for these data. Stepwise regression analysis was performed to acquire the inverse solutions for the data, during which characteristic, significantly represented spectral bands were accurately screened out from the complex spectral data based on the correlation coefficient values, r, and the determination coefficient, *R*^*2*^, in the regression equation.

#### 2.4.3 Single-modality model establishment

Quantitative predictive models for zeaxanthin were constructed using the preprocessed spectral data as the input. Comparative analysis was performed on the modeling effects of the characteristic spectral bands and on all of the spectral bands (365–970 nm) to determine the independent variable. Then, the zeaxanthin contents of the of chili pepper exocarps were used as the dependent variable, and models were generated using random forest (RF), back propagation (BP), one-dimensional convolutional neural network (1D CNN), and partial least squares (PLS) methods to predict zeaxanthin content.

#### 2.4.4 MCSF modeling

**Fig. 1:**
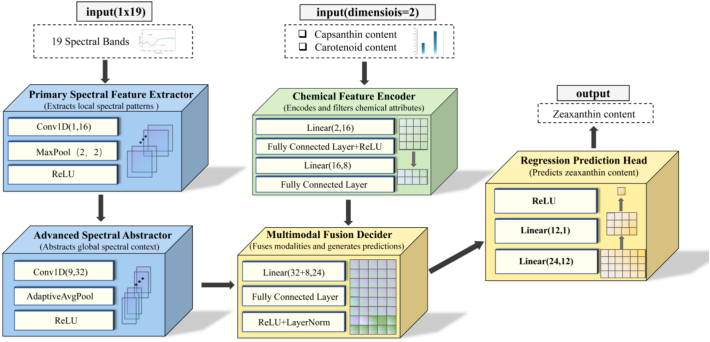
Overview of the MCSF model encoder modularity.

To enhance the accuracy of the zeaxanthin content predictions, an MCSF model was constructed based on the optimal single-modal model. Additionally, two different types of data, namely the spectral information of the chili pepper exocarp and the chemically determined content of capsanthin and total carotenoids, were used for model learning. The 1D CNN model was then used for feature extraction of the preprocessed spectral data. It gradually captured local features and abstract features in the spectrum by multi-layer convolution and pooling and finally output a set of feature vectors representing spectral features. The capsanthin and total carotenoid contents, as chemically determined, were processed using a multilayer perceptron. First, they were projected into a 16-dimensional space and activated using a rectified linear unit function, and then compressed into 8-dimensional feature vectors to capture the nonlinear relationships between the chemical indicators. The two sets of feature vectors extracted were spliced and input into the fusion module and regression prediction head, and the complex relationships between these input features and zeaxanthin content were then learned through data training, finally achieving an accurate prediction of zeaxanthin content, as shown in Fig. 1.

To further investigate the feature-level mechanisms underlying the predictive performance of the MCSF model, we extracted the feature representations from both the spectral-only model (M-Spectral) and the MCSF model prior to the final prediction layer and projected them into two-dimensional space using t-SNE for visualization.

#### 2.4.5 Model interpretability analysis

To quantitatively evaluate the contribution of chemical data to predictive performance, a systematic ablation experiment was conducted. Five models were constructed with different input configurations: M-Spectral using only spectral data, M-Chemical using only the two chemical indicators (capsanthin and total carotenoid content), M-S + R using spectral data combined with random noise, M-S + N using spectral data combined with slightly perturbed chemical data (adding 5% Gaussian noise), and M-MCSF using spectral data combined with authentic chemical data. All models were trained under the same hyperparameters, and their performance was compared on the test set.

To investigate the feature-level mechanisms underlying the predictive performance of the MCSF model, feature representations were extracted from both the spectral-only model (M-Spectral) and the MCSF model prior to the final prediction layer. These feature vectors were then projected into two-dimensional space using t-distributed stochastic neighbor embedding (t-SNE) for visualization, allowing qualitative assessment of the feature space structure and its alignment with genetic background.

To further validate the robustness of the MCSF model, a sensitivity analysis was conducted to examine how varying the correlation between the chemical features and the target variable influences prediction accuracy. Specifically, synthetic capsanthin values with different correlation coefficients (ranging from 0.2 to 0.95) relative to the true zeaxanthin content were generated, while total carotenoid content was kept unchanged. The performance of the MCSF model was then evaluated on the test set under each condition.

#### 2.4.6 Comprehensive red index (CRI) construction

In this study, a modified MCSF model, namely the MCSF model with feature replacement (designated MCSF-FR), was employed. The MCSF-FR model retained the original MCSF network architecture and hyperparameter settings but was retrained using the CRI as a substitute for the measured value of capsanthin content. Based on the spectral band reflectance ratios, four distinct indices were calculated: the red-to-green ratio (R/G), intended to enhance the dominance of red over green; the red-to-blue ratio (R/B), intended to accentuate red relative to blue; the normalized red-green index (NRGI, NRGI = (R − G)/(R + G)), which standardized the difference between red and green; and the red purity index (RPI, RPI = R/(R + G + B)), representing the proportion of the red component in the total brightness. The CRI was subsequently formulated as a proxy feature for capsanthin content by integrating these indices using the following weighted formula: CRI = 0.4 × (R/G) + 0.3 × (R/B) + 0.2 × NRGI +0.1 × RPI.

#### 2.4.7 Dataset division and model assessment

The 159 chili pepper powder samples tested were assigned to a calibration set (*n* = 126, encompassing a validation set) and a test set (*n* = 33) at a ratio of 8:2. Dataset division was completed by stratified sampling, and the divided datasets were tested for spectral features, chemical features, and distribution consistency of the target variables, to ensure the rationality of the division. To further evaluate the robustness of the model, the calibration set was further divided into a training set and a validation set by a leave-one-out cross-validation (LOOCV) method (Mello et al., 2025). Model accuracy was comprehensively appraised using: *R*^*2*^ for the training set (*Rt*^*2*^); *R*^*2*^ for the calibration set (*Rc*^*2*^); *R*^*2*^ for the validation set (*Rv*^*2*^); *R*^*2*^ for the prediction set (*Rp*^*2*^); root mean square error for the training set (*RMSEt*); *RMSE* for the calibration set (*RMSEc*); *RMSE* for the validation set (*RMSEv*); *RMSE* for the prediction set (*RMSEp*); residual prediction deviation (*RPD*); and a 1:1 line based on the model evaluation index system. Of these, *RPD* ≥ 3 indicated the reliable predictive ability of the model (Sinelli et al., 2008), while *RPD* = 2.4–3 denoted an inadequate model performance, only suitable for general prediction. An ideal model should have high *R*^*2*^ and *RPD* and low *RMSE*. The 1:1 line analysis visualized the deviation of the model from the theoretical value of y = x by plotting a scatter plot of the predicted and measured values.

#### 2.4.8 External validation

To further evaluate the generalization capability of the proposed model, an independent external validation set consisting of 39 samples was collected. Similarly, for the independent external validation set, the coefficient of determination (*R*_*Ext*_^*2*^), root mean square error (*RMSE*_*Ext*_) were calculated to evaluate model generalization performance.

All models were trained and tested under identical hardware conditions (NVIDIA GeForce RTX 3070). Parameter counts were calculated based on trainable parameters. Training time was recorded as the total time for a single training pass on the full training set, and inference time was measured as the average prediction time per sample (including GPU synchronization warm-up).

Multiple machine learning algorithms were integrated during the study, and the statistical analysis and visual expression of the multi-dimensional performance indicators were accomplished using the software platforms Python v.3.11 and Origin 2022.

$$R^{2}=\frac{\sum_{i=1}^{n}{\left({\hat{y}}_{i}-{\bar{y}}_{i}\right)}^{2}}{\sum_{i=1}^{n}{\left(y_{i}-{\bar{y}}_{i}\right)}^{2}}$$

$$\mathrm{RMSE}=\sqrt{\frac{\sum_{i=1}^{n}{\left(y_{i}-{\hat{y}}_{i}\right)}^{2}}{n}}$$

$$\mathrm{RPD}=\frac{\mathrm{SD}}{\mathrm{RMSE}}$$

where ${\hat{y}}_{i}$, $\bar{y}$, and $y_{i}$ stand for the predicted value, mean, and measured values of the content, respectively. n is the number of samples $\left(i=1,2,3\ldots n\right)$. *SD* is the standard deviation.

## 3 Results and discussion

### 3.1 Spectral features of the exocarp of chili peppers

**Fig. 2:**
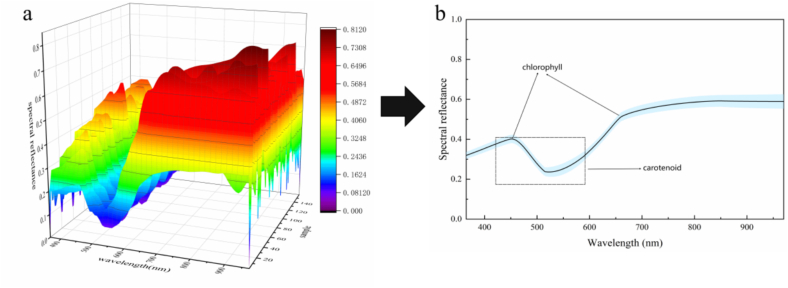
Spectral information on the chili pepper exocarp. (a) Spectral reflectance of chili pepper exocarp (*n* = 159), and (b) mean spectral reflectance of chili pepper exocarp.

Fig. 2a shows the spectral reflectance results of the 159 chili pepper exocarp samples in the wavelength range 365–970 nm, revealing variations in the light absorption properties of samples across different wavelengths. In the visible region (400–700 nm), the difference in spectral reflectance of chili pepper powder was the most significant, and the spectral data of the samples had the greatest variation intensity. Variations in reflectance around the 450 nm and 670 nm wavelengths were mainly attributed to changes in chlorophyll levels (Fig. 2b) (Ou et al., 2005; Shah et al., 2016).The low chlorophyll content of chili pepper fruit at full ripening (Moser & Matile, 1997) probably results in weakened absorption of blue and red light, leading to increased reflectance. In the near-infrared region (700–900 nm), the reflectance tended to be flat, probably due to the extremely weak water absorption effect of these dry powder samples (Rahman et al., 2018). A reflectance dip in the plot of mean spectral reflectance occurred in the region of 520 nm, and was ascribed to the combined action of multiple carotenoids (Zhou, Wang, et al., 2017). The absence of observable characteristic peaks significantly correlated with zeaxanthin content in the figure thus necessitates the establishment of a quantitative relationship between the content and the spectral features using multivariate analysis.

### 3.2 Statistical analysis of substance content

**Fig. 3:**
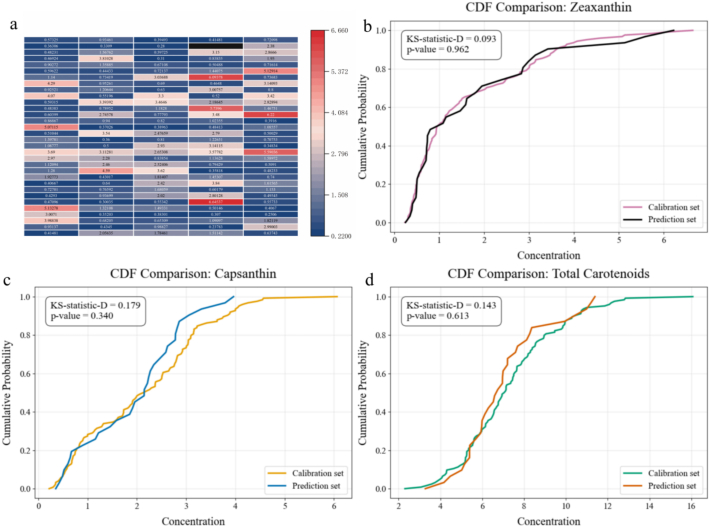
Distribution of substance content of chili peppers. (a) Heat map of zeaxanthin content of the samples, and cumulative distribution diagrams of (b) zeaxanthin, (c) capsanthin, and (d) total carotenoid contents of the calibration/prediction set. Note: The vertical axis of cumulative distribution diagrams represents the cumulative probability of the content less than or equal to the corresponding horizontal axis.

The zeaxanthin contents of the 159 samples were compared, and the zeaxanthin content was shown to be 0.237–6.64 g/kg in the calibration set, covering the minimum and maximum values of the whole sample set, with a coefficient of variation (CV) of 87.88% (Table S1). The zeaxanthin content was 0.25–6.22 g/kg in the prediction set, with a CV of 95.84%, and varied significantly (Table S2, Fig. 3a). The Kolmogorov-Smirnov (KS) test was used to calculate the *D* statistic and corresponding *p*-value (significance threshold α =0.05) to assess the distribution similarity between the calibration set and the prediction set. The results showed *D* = 0.093 and *p* = 0.962, for the target variable (zeaxanthin content) (Fig. 3b). Regarding the all-band (365–900 nm) reflectance, *D* = 0.096–0.239 and *p* = 0.090–0.952 (Table S3). Moreover *D* = 0.179 and *p* = 0.340 (Fig. 3c), and *D* = 0.143 and *p* = 0.613 were achieved in terms of capsanthin content and total carotenoid content, respectively (Fig. 3d).

All of the KS test results failed to reject the null hypothesis (*p* > 0.05), and the calibration set and the prediction set showed no statistically significant differences in the distribution of zeaxanthin content, all-band reflectance, and total capsanthin and carotenoid contents. This suggests a reasonable dataset division of the calibration set and the prediction set, which can therefore be used for subsequent model performance comparisons to establish a strongly representative quantitative analytical model for zeaxanthin with the potential for generalized use (Gao et al., 2022).

### 3.3 Screening and analysis of characteristic spectral bands

**Fig. 4:**
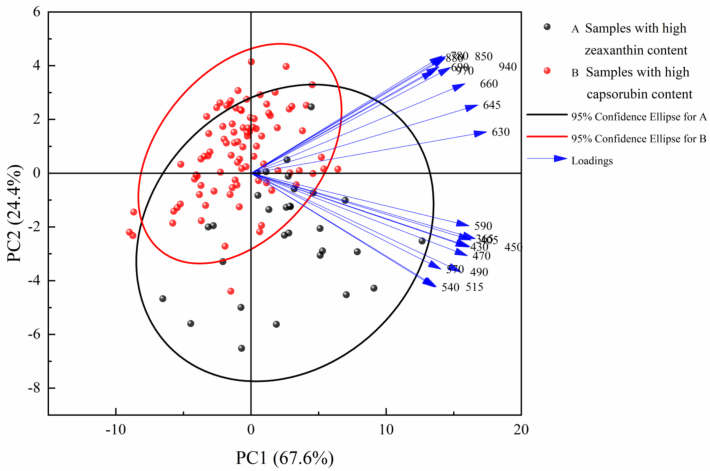
PCA on spectral reflectance.

The reflectance data of the 159 samples across all spectral bands were subjected to the Kaiser-Meyer-Olkin (KMO) test and Bartlett's sphericity test. The results revealed that *KMO* = 0.828 (>0.7) and *p* < 0.001, indicating that the data met the fundamental requirements of PCA. The first two principal components (PCs) accounted for 92.0% of the total variance (PC1 = 67.6%, PC2 = 24.4%) (Fig. 4), and effectively characterized the core information of the original 19 spectral wavelengths (365–970 nm) (Tian & Wang, 2022) A composite score was calculated based on the first two PCs and the reflectance values at various spectral wavelengths, and five characteristic wavelengths (405, 540, 570, 645 and 970 nm) (*r* = 0.999, *R*^*2*^ = 0.999) were screened out by reverse stepwise regression analysis, based on the quantitative relationship between the composite scores of the PCs and the spectral reflectance, achieving efficient dimensionality reduction while retaining the original variable information (a spectral reflectance of 92%). All-band regression analysis also showed an excellent fit to the data (*r* = 1.000, *R*^*2*^ = 1.000). By these means, predictive models for zeaxanthin content of chili pepper exocarp were created with spectral reflectance in the five characteristic wavelength bands and all spectral bands as the dependent variables. This PCA-based wavelength selection method is applicable to various types of spectral data, as it does not depend on the specific data type (Jia et al., 2022); it has been successfully applied to hyperspectral, multispectral, near-infrared, and Raman spectroscopy (Gao et al., 2024; Liu et al., 2021; Stradling et al., 2025).

### 3.4 Comparison of single-modality models

#### 3.4.1 Analysis of the model established based on all spectral bands

According to the model based on all spectral bands (Table 1), the RF, 1D CNN, BP, and PLS algorithms exhibited significant differences in their predictive accuracy for zeaxanthin. The nonlinear algorithm 1D CNN model had the optimal predictive accuracy, with higher *Rt*^*2*^ (=0.9742) and *Rv*^*2*^ (=0.7638) than those of the other models, as well as the lowest *RMSEt* (=0.1996) and *RMSEv* (=0.7169). The BP model showed an excellent *Rt*^*2*^ (=0.9422), but its *Rv*^*2*^ was only 0.7362, and its *ΔR*^*2*^ (=0.2060) indicated a severe risk of overfitting. The PLS model showed the lowest predictive ability (*Rt*^*2*^ = 0.6782, *Rv*^*2*^ = 0.4881) due to the limitations of its linear assumptions, whereas its *ΔR*^*2*^ (=0.1901) was relatively small, showing good generalization ability. These results hint that the nonlinear algorithm 1D CNN is more suitable for the analysis of zeaxanthin spectral features.

**Table 1:** Comparison results of the validation set among the four models under different bands.

Spectral band types	Model	Rt^2^	Rv^2^	RMSEt	RMSEv	ΔR^2^
All spectral bands	RF	0.7966	0.5532	0.6392	1.0111	0.2434
	1D CNN	0.9742	0.7638	0.1996	0.7169	0.2104
	BP	0.9422	0.7362	0.3388	0.8119	0.2060
	PLS	0.6782	0.4881	0.8037	1.1210	0.1901

Characteristic spectral band	RF	0.7562	0.5426	0.7001	0.9720	0.2136
	1D CNN	0.8351	0.6701	0.5752	0.8964	0.1650
	BP	0.7821	0.5866	0.6606	1.0722	0.1955
	PLS	0.6566	0.4529	0.8310	1.1671	0.2037

#### 3.4.2 Influence of screening of characteristic spectral bands on the model

In the characteristic spectral bands, the 1D CNN model still achieved the highest *Rt*^*2*^ (=0.8351) and *Rv*^*2*^ (=0.6701), but a decrease of 12.27% was observed in the validation set compared with all spectral bands, and its *ΔR*^*2*^ (=0.1650) was lower than that in all spectral bands (*ΔR*^*2*^ = 0.2104). This indicates that band reduction mitigates the risk of overfitting, but that the predictive accuracy of the model declines owing to the loss of partial spectral information. For the RF model, the *RMSEv* decreased from 1.0111 in all spectral bands to 0.9720 (with an improvement of 3.86%) in the characteristic spectral bands, and the model showed stronger anti-redundancy and anti-interference capabilities, as well as enhanced generalization ability.

#### 3.4.3 Independent verification of the prediction set and model optimization

**Fig. 5:**
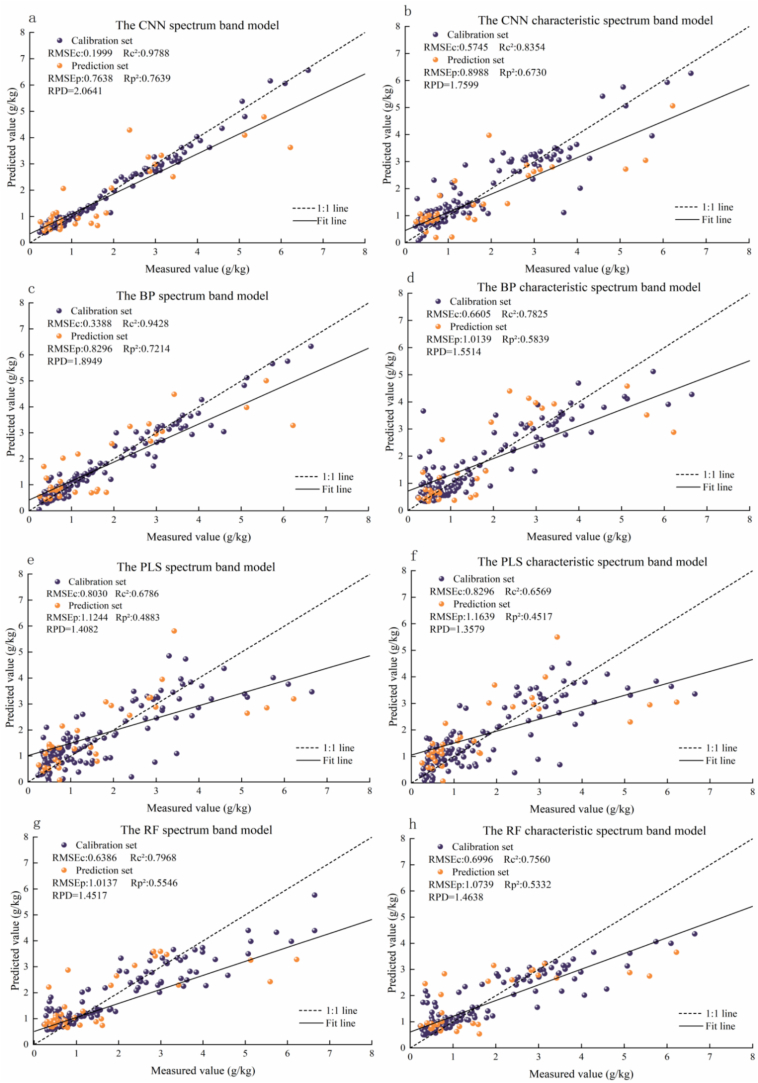
Prediction results for zeaxanthin in chili peppers obtained by different models: (a) 1D CNN, (c) BP, (e) PLS, and (g) RF prediction models based on all spectral bands; and (b) 1D CNN, (d) BP, (f) PLS, and (h) RF prediction models based on characteristic spectral bands.

The prediction set was independently verified, and it was discovered that the superiority (*Rp*^*2*^ = 0.7639) of the 1D CNN model in all-band data was better than that of the other models, basically in line with its excellent performance in the validation set and implying that the 1D CNN model is favorable for generalization. The fitting curve of the 1D CNN model was closest to the y = x (1:1) line, and its *RPD* > 2.0 denoted favorable prediction capability (Fig. 5). The performance (*Rp*^*2*^ = 0.7639) was comparable to that of hyperspectral CNN models predicting total carotenoids (*Rp*^*2*^ = 0.83–0.86) or total anthocyanins (*Rp*^*2*^ = 0.83–0.87) (Bao et al., 2025; Huang et al., 2024). However, because the above two experiments predicted the content of total flavonoids or total anthocyanins rather than a specific, single substance, the Rp^2^ values (0.83–0.86) in these two studies are higher than that of the present study (0.7639). Furthermore, the *Rp*^*2*^ value in this study is comparable to or higher than those for predicting other individual compounds, such as salicinoids (*R*^*2*^ = 0.57) (Couture et al., 2016) and cyanidin 3-rutinose in Michelia crassipes tepals (*R*^*2*^ = 0.72) (Xiao et al., 2024). Therefore, the 1D CNN model was established as the optimal single-modal model for the prediction of zeaxanthin contents.

The moderate performance of the 1D CNN model can be attributed to spectral multicollinearity, similar to the prediction of the aforementioned individual compounds, which also suffer from weak or overlapping spectral features. This spectral multicollinearity prevents 1D CNN models from distinguishing the contributions of zeaxanthin and capsanthin, leading to inflated variance and overfitting (Claret et al., 2016; Dertli et al., 2024). As demonstrated in milk protein prediction (Eskildsen et al., 2016) and chili pepper LAI prediction (Zhang, Wang, et al., 2025), models relying on indirect relationships can fail when covariance structures change. Here, the 1D CNN achieved only moderate performance (*Rp*^*2*^ = 0.7632) because it risked capturing indirect covariance between zeaxanthin and capsanthin.

### 3.5 Assessment of the constructed multimodal model

#### 3.5.1 Comparison between MCSF and 1D CNN models

According to the model generalization ability and robustness assessments, the MCSF model outperformed the 1D CNN model across the board (Table 2). In all spectral bands, the MCSF model achieved higher predictive accuracy in the validation set (*Rv*^*2*^ = 0.9262), significantly better than that of the 1D CNN model (*Rv*^*2*^ = 0.7638). In addition, the MCSF model had close predictive accuracy in the training set and the validation set (*ΔR*^*2*^ = 0.0563), suggesting better generalization ability, whereas the 1D CNN model showed a strong tendency to overfit, and a large difference in predictive accuracy between the training set and the validation set (*ΔR*^*2*^ = 0.2104). This robustness advantage was also reflected in the *RMSE* indicators. The 1D CNN model had an elevation of 259.2% in the *RMSEv* in the validation set compared to that in the training set, while the MCSF model showed an increase of only 70.46%. Compared with the all spectral bands performance, the MCSF model had a decrease of only 1.33% in *Rv*^*2*^ in the characteristic spectral bands, whereas the 1D CNN model displayed a reduction of 12.0% in *Rv*^*2*^. The MCSF model reduced the spectral information loss resulting from band reduction by fusing multimodal features and still possessed high predictive accuracy in the five characteristic spectral bands. This minimal loss highlights the advantage of band selection for the MCSF model. Band selection reduces model complexity and suppresses overfitting, as shown in studies where it improved *RPD* by 12% (Chen et al., 2024) or increased *Rp*^*2*^ from 0.91 to 0.93 (Qiao et al., 2026). However, its effectiveness depends on the target compound's spectral feature intensity (Zenil et al., 2025), particularly when the spectral features are weak, band selection can result in noticeable performance degradation, as seen in chlorophyll *b* (*Rv*^*2*^ from 0.58 to 0.52) and dietary fiber prediction (Zhang et al., 2023; Zhang et al., 2024). This limitation arises from the spectral signals themselves, not from the band selection strategy (Couture et al., 2016).

**Table 2:** The MCSF/MCSF-FR model and 1D CNN model in the calibration and validation sets.

Spectral band types	Model	Rt^2^	Rv^2^	RMSEt	RMSEv
All spectral bands	1D CNN	0.9742	0.7638	0.1996	0.7169
	MCSF	0.9825	0.9262	0.2258	0.3849
	MCSF-FR	0.9673	–	0.2560	–

Characteristic spectral band	1D CNN	0.8351	0.6701	0.5752	0.8964
	MCSF	0.9911	0.9139	0.1033	0.4156
	MCSF-FR	0.9855	–	0.2205	–

MCSF has shown significant potential in raising the performance of spectral detection models in recent years, especially in medical imaging and simple component analysis (Ding et al., 2025; Zhang, Wang, et al., 2025; Zhang, Li, et al., 2025). In this study, MSI integrating chemical features was applied for the detection of zeaxanthin for the first time, and the MCSF model was established, which effectively enhanced the detection accuracy and robustness of the model, consistent with previous research findings.

#### 3.5.2 Prediction set performance of the MCSF model

**Fig. 6:**
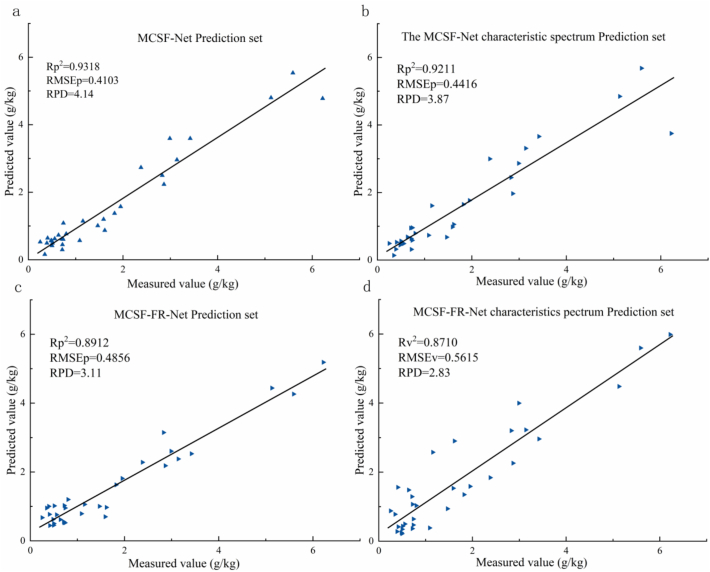
Results of the MCSF/MCSF-FR model in the validation and prediction sets in all spectral bands and the characteristic spectral bands. Results of the (a) MCSF and (c) MCSF-FR models in all spectral bands and of the (b) MCSFF and (d) MCSF-FR models in the characteristic spectral bands.

The results of the MCSF model in the prediction set (Fig. 6) showed that the MCSF model produced excellent performance regarding both all spectral bands and the characteristic spectral bands: the *Rp*^*2*^ reached 0.9318 and 0.9211, respectively, basically consistent with *Rv*^*2*^. In addition, the *RPD* was 4.14 in all spectral bands and 3.87 in the characteristic spectral bands, both exceeding 3.0, implying that the model has excellent predictive ability and practicability. In the five characteristic spectral bands, a drop of only 1.15% was noted in the *Rp*^*2*^ of the MCSF model compared with that in all spectral bands, which was smaller than the decrease (11.9%) in the 1D CNN model under the same conditions. For the MCSF model, using five characteristic bands achieved an *Rp*^*2*^ of 0.9211 (a 1.15% decline), while reducing parameters by 49.9% and improving theoretical computational efficiency by ∼80%, making it suitable for portable devices while maintaining high-throughput screening capability (*Rp*^*2*^ > 0.92). The statistical test results further support the reliability of the MCSF model: the mean difference between the predicted value and the measured value was 0.1490 g/kg, and the *t*-test value (2.170) was less than *t*_*0.01*_ (2.744), demonstrating no significant difference between the two and proving the accuracy of the model output.

#### 3.5.3 Sensitivity analysis of chemical feature correlation

As illustrated in Fig. S3, the test set *Rp*^*2*^ steadily increased from 0.25 to 0.90 as the correlation between the simulated capsanthin and zeaxanthin increased. This trend demonstrates that the MCSF model effectively leverages strongly correlated prior information without suffering from performance degradation or overfitting. Conversely, stronger correlation provides more accurate constraints, enabling the model to learn the residual spectral variations associated with zeaxanthin more precisely. This analysis validates the necessity of incorporating chemical features and confirms that the MCSF model remains robust across a wide range of correlation strengths, further underscoring its practical utility.

This robust performance can be attributed to the introduction of measured capsanthin content as an independent prior, which decouples the contributions of zeaxanthin and capsanthin. This transforms a complex two-variable disentanglement problem into a simpler conditional single-variable estimation, stabilizing parameter estimation and improving generalization.

#### 3.5.4 Ablation experiment

We designed an ablation experiment to evaluate the contribution of chemical data(Table S4). The spectral-only model (M-Spectral) achieved an *Rp*^*2*^ of 0.7632, while the chemical-only model (M-Chemical) achieved only 0.4425. Replacing the chemical data with random noise (M-S + R) reduced performance to 0.5750, and using perturbed chemical data (M-S + N) yielded 0.8253, both significantly lower than the MCSF model using authentic chemical data (0.9318). These results demonstrate that the enhancement in predictive accuracy originates from the specific biochemical information carried by the chemical data, rather than from increased input dimensionality.

#### 3.5.5 Feature space visualization via t-SNE

In the feature space of the spectral-only model (Fig. S2a), samples exhibited a continuous, gradient-like distribution without forming clear class boundaries, and the separation did not align with the actual genetic background (3:1 segregation ratio). In contrast, the fused feature space of the MCSF model (Fig. S2b) showed a fundamental improvement: samples formed two distinct clusters that aligned precisely with the expected segregation ratio, with an orderly gradient distribution of zeaxanthin content within each cluster. These results demonstrate that incorporating chemical data enables a hierarchical feature learning strategy—first classifying samples by genetic background, then performing regression—substantially enhancing model robustness and interpretability.

#### 3.5.6 Grad-CAM analysis of spectral weight adaptation

Grad-CAM visual analysis has been employed to visualize input variable weights in complex CNN architectures (Xu et al., 2024). The results of this study showed that the MCSF model had significantly reduced activation intensity in the 430–500 nm band (highly overlapping with the strong absorption region of capsanthin) (Fig. S1), hinting that the MCSF model not only actively suppresses the spectral response of the overlapping band after introducing information on capsanthin content, but also realizes the transition from passive noise reduction to active decoupling by introducing total carotenoids (a global content constraint), significantly improving the interference cancellation effect. The adaptive adjustment ability of spectral weights may be a crucial contributor to the improvement of MCSF predictive accuracy.

#### 3.5.7 Computational complexity analysis

The comparisons of the complexity between the MCSF and 1D CNN were performed (Table S5). The results showed that the MCSF model achieved a substantial improvement in predictive performance (*Rp*^*2*^ was increased from 0.7639 to 0.9318) with an 80.5% increase in parameter count (from 2177 to 3929) and an inference time of 0.364 ms per sample, which remained well below 0.5 ms. These results demonstrate that the MCSF model reaches an excellent balance between complexity and performance, fully satisfying the requirements for real-time detection applications.

#### 3.5.8 Performance of the MCSF-FR model

When the RGB band-derived CRI was introduced as the input feature, in place of the measured value of capsanthin, the MCSF-FR model showed decreases in *Rt*^*2*^ (from 0.9825 to 0.9673) (Table 2), *Rp*^*2*^ (from 0.9318 to 0.8912), and *RPD* (from 4.14 to 3.11) in all spectral bands (Fig. 6c). Regarding the characteristic spectral bands, the model *Rt*^*2*^ rose (from 0.9742 to 0.9855) (Table 2) with falling *Rp*^*2*^ (from 0.9211 to 0.8659) and *RPD* (from 3.87 to 2.83) (Fig. 6d). These results show that the model still possesses good predictive ability. CRI was selected as a proxy for capsanthin because, in red and orange mature peppers, capsanthin is the dominant pigment and its content correlates directly with fruit redness. In contrast, alternative indices are less suitable: NDVI primarily reflects chlorophyll (Joiner et al., 2018), PRI shows weak specificity for capsanthin (Gamon et al., 2023; Magney et al., 2016), ARI targets anthocyanins, and red-edge parameters require high spectral resolution (Vanoli et al., 2020). Thus, CRI offers the advantages of being highly correlated with capsanthin, computationally simple, and well-suited for high-throughput screening in breeding program.The minor drop in model performance is attributable to the fact that CRI cannot fully represent the measured value of capsanthin. Nevertheless, studies on wheat wet gluten (*RPD* = 2.70), peanut phenolics (*RPD* = 2.77–2.88), and rice nutritional quality (*RPD* = 3.62 and 3.46)demonstrate that models with *RPD* values in the range of 2.70–3.62 meet the requirements for practical production applications (Haruna et al., 2023; John et al., 2022; Lai et al., 2026). By showing that CRI is an effective substitute for capsanthin and that the MCSF model architecture itself possesses strong robustness, this strategy enables an optimized detection workflow at the cost of only negligible precision loss.

In conclusion, the MCSF model achieves a good balance among band number, predictive accuracy, and computational efficiency under the characteristic spectral bands. When using CRI as a substitute for the measured value of capsanthin as the input feature, the MCSF-FR model exhibits the optimal overall performance in all spectral bands.

#### 3.5.9 External validation

The external validation results demonstrated strong performance (Fig.S4). For the full-spectrum model, *R*_*Ext*_^*2*^ = 0.9079, *RMSE*_*Ext*_ = 0.4588, and *RPD* = 3.34; for the characteristic spectral bands, *R*_*Ext2*_ = 0.8783, *RMSE*_*Ext*_ = 0.5275, and *RPD* = 2.90. The external validation results demonstrated strong performance, confirming that the model exhibits cross-generational stability within pepper genetic populations that accumulate zeaxanthin, thus supporting its application in high-zeaxanthin breeding material screening. Despite its effectiveness under the conditions of this study, the applicability of CRI as a substitute for capsanthin is subject to certain constraints, including genotypic variation, immature stages where chlorophyll dominates, and processing methods that may alter pigment composition (Deli et al., 2001; Mohd Hassan et al., 2019; Rodríguez-Burruezo et al., 2010; Shah et al., 2013). However, these limitations do not compromise the validity of the CRI-based approach in this study, which focuses on red and orange mature peppers for high-zeaxanthin breeding selection. Furthermore, the model can be transferred via transfer learning to other crops that share the same carotenoid biosynthetic pathway as pepper, such as tomato, goji and marigold (Liu et al., 2015; Shahverdi et al., 2026; Sun et al., 2023). In future work, model robustness against field environmental variations could be further enhanced by integrating environmental covariates and expanding the training dataset to include samples from diverse growing seasons and locations.

## 4 Conclusion

An efficient predictive method for the zeaxanthin content of chili peppers was constructed based on MSI technology. The 1D CNN model was shown to be the optimal single-modal model (*Rp*^*2*^ = 0.7639, *RPD* = 2.0641) as revealed by the comparison of the four tested algorithms. To address the problem of spectral interference of the target chemical substances, an MCSF model integrating spectral and chemical features was constructed, with significantly enhanced predictive performance. The predictive accuracy of the MCSF model using characteristic bands (*Rp*^*2*^ = 0.9211) was 20.58% higher than that of the 1D CNN single model in the full-spectrum bands, with an *RPD* value of 3.87, better than that of 1D CNN model (2.06). The MCSF-FR model achieved an optimal balance between spectral data volume and predictive accuracy. By substituting the CRI for the measured capsanthin content, the MCSF-FR model still used the full spectrum and remained a high-quality model. Its *Rp*^*2*^ was 0.8912 and *RPD* was 3.11. The proposed method enables cross-modal feature complementation, solves the problem of spectral multicollinear interference, and provides a novel approach for the efficient spectrum-based detection of phytochemicals affected by their multicollinearity of absorption peaks.

## CRediT authorship contribution statement

**Wei Liang:** Writing – original draft, Methodology, Data curation. **Wei Gao:** Investigation. **Xingna Lv:** Formal analysis. **Jinxiang Zhao:** Data curation. **Xuechun Tian:** Data curation. **Shuaitao Di:** Data curation. **Qiang Li:** Visualization. **Dongfang Zhang:** Formal analysis. **ShuangXia Luo:** Writing – review & editing, Supervision, Resources, Funding acquisition, Conceptualization. **XuePing Chen:** Resources, Formal analysis, Conceptualization.

## Declaration of competing interest

The authors declare that we have no known competing financial interests or personal relationships that could have appeared to influence the work reported in this paper.

## Data Availability

The datasets and the main model code are available online at https://github.com/liang-wei-tian/Chili-Peppers-Zeaxanthin.
