# Supplementary material for: Multispectral imaging for zeaxanthin content in the exocarp of chili peppers
Source: Food Chem X. 2026 May 18;36:103992. doi: 10.1016/j.fochx.2026.103992 (PMC13213780; doi:10.1016/j.fochx.2026.103992)
Supplement: Supplementary file 1 — Supplementary Information. [file mmc1.docx]

**Table S1**: Substance content statistics for the calibration set

| Calibration set | Capsanthin | Total carotenoids | Zeaxanthin |
| --- | --- | --- | --- |
| 1 | 2.147073954 | 5.931608181 | 0.414806397 |
| 2 | 2.511852066 | 7.663832081 | 0.93136506 |
| 3 | 0.433588529 | 6.694171707 | 3.98838382 |
| 4 | 1.000685888 | 6.533664024 | 3.007097325 |
| 5 | 1.717979136 | 10.71271634 | 5.132780413 |
| 6 | 2.522574047 | 7.171928196 | 0.470956431 |
| 7 | 3.100887249 | 7.443060234 | 0.429299342 |
| 8 | 3.04004505 | 8.139793285 | 0.727011068 |
| 9 | 1.283874436 | 4.305941135 | 0.40666506 |
| 10 | 4.549758227 | 12.84722931 | 1.927729441 |
| 11 | 0.35 | 3.07 | 1.28 |
| 12 | 4.047508533 | 11.00537197 | 1.12093609 |
| 13 | 0.86 | 6.17 | 2.97 |
| 14 | 0.68 | 6.87 | 3.69 |
| 15 | 3.090007864 | 8.619916912 | 1.087767598 |
| 16 | 2.927410648 | 8.950530077 | 1.397809465 |
| 17 | 1.815037447 | 4.947881314 | 0.510442859 |
| 18 | 1.321635002 | 9.784677114 | 5.071154556 |
| 19 | 3.034959202 | 8.192649939 | 0.868667627 |
| 20 | 1.572107316 | 5.351860623 | 0.603992701 |
| 21 | 3.844010774 | 8.927247904 | 0.483826941 |
| 22 | 2.680855735 | 6.816770015 | 0.593147694 |
| 23 | 0.84 | 7.64 | 4.07 |
| 24 | 2.002789822 | 6.502339802 | 0.925214603 |
| 25 | 0.9 | 8.54 | 4.29 |
| 26 | 2.66 | 8.4 | 1.14 |
| 27 | 3.41967171 | 9.051135056 | 0.596221177 |
| 28 | 2.761638091 | 8.1858307 | 0.90271607 |
| 29 | 2.531971203 | 6.464235205 | 0.469235628 |
| 30 | 2.799865213 | 7.139048504 | 0.482313331 |
| 31 | 1.985331255 | 5.607777861 | 0.363060735 |
| 32 | 2.292092886 | 6.278653554 | 0.573246011 |
| 33 | 0.745782613 | 4.63914849 | 2.056346501 |
| 34 | 1.621804058 | 4.841854683 | 0.43450107 |
| 35 | 2.752974077 | 7.505863636 | 0.682049716 |
| 36 | 1.869633216 | 5.188446714 | 0.352031886 |
| 37 | 3.092503822 | 9.467910594 | 1.321078348 |
| 38 | 1.709590218 | 4.292797517 | 0.30034684 |
| 39 | 2.9161422 | 8.30833246 | 0.936989457 |
| 40 | 4.256353103 | 10.48684627 | 0.765924466 |
| 41 | 3.16 | 7.84 | 0.64 |
| 42 | 2.056886076 | 5.085327792 | 0.43016879 |
| 43 | 1.19 | 8.96 | 4.59 |
| 44 | 0.69 | 5.26 | 2.46 |
| 45 | 0.7 | 5.2 | 2.28 |
| 46 | 0.352443459 | 5.349755982 | 3.112812534 |
| 47 | 1.96 | 5.6 | 0.5 |
| 48 | 2.47 | 5.72 | 0.36 |
| 49 | 0.74 | 6.77 | 3.54 |
| 50 | 2.372755107 | 6.588971763 | 0.370255503 |
| 51 | 2.81 | 7.11 | 0.94 |
| 52 | 0.784634353 | 6.057409609 | 2.745780249 |
| 53 | 2.645218904 | 6.948480814 | 0.789523603 |
| 54 | 0.908306991 | 7.045642007 | 3.39392404 |
| 55 | 3.044010992 | 8.029153668 | 0.551958524 |
| 56 | 3.63012671 | 10.06907345 | 1.206444321 |
| 57 | 2.505982155 | 7.375918746 | 0.9526057 |
| 58 | 2.381656222 | 6.946310935 | 0.734188262 |
| 59 | 3.00039089 | 7.880345342 | 0.444329626 |
| 60 | 4.000682871 | 10.73452044 | 1.358848263 |
| 61 | 0.750305683 | 7.45656281 | 3.810279583 |
| 62 | 2.826523958 | 9.465516901 | 1.56761696 |
| 63 | 1.90672311 | 5.438918252 | 0.330904529 |
| 64 | 3.627949654 | 9.93817528 | 0.934606623 |
| 65 | 4.440727644 | 12.32495115 | 1.784614277 |
| 66 | 2.502786014 | 7.653109658 | 0.988271281 |
| 67 | 3.756010232 | 9.85825034 | 0.653086473 |
| 68 | 1.734359942 | 5.644056192 | 0.38301331 |
| 69 | 4.569042239 | 11.86130031 | 1.493310791 |
| 70 | 2.927277019 | 7.656806511 | 0.553416773 |
| 71 | 0.51 | 4.07 | 2.02 |
| 72 | 0.407750332 | 3.751860135 | 1.680591901 |
| 73 | 0.68 | 5.2 | 2.42 |
| 74 | 6.063669102 | 16.06901907 | 1.814068231 |
| 75 | 0.54 | 6.76 | 3.62 |
| 76 | 0.302375809 | 3.864044345 | 2.524061943 |
| 77 | 3.150868602 | 8.553554809 | 0.83854349 |
| 78 | 0.621757773 | 5.536305145 | 2.653080559 |
| 79 | 0.67 | 5.86 | 2.93 |
| 80 | 4 | 9.85 | 0.81 |
| 81 | 0.581381085 | 5.260782078 | 2.476593439 |
| 82 | 2.792487614 | 7.507510456 | 0.389632873 |
| 83 | 2.13 | 5.9 | 0.82 |
| 84 | 2.816445004 | 8.519225928 | 0.777926999 |
| 85 | 3.885275228 | 10.27636498 | 1.182802817 |
| 86 | 0.586956319 | 6.418176996 | 3.464601336 |
| 87 | 0.33 | 6.15 | 3.3 |
| 88 | 3.16 | 7.36 | 0.63 |
| 89 | 1.92 | 5.18 | 0.69 |
| 90 | 1.130786614 | 6.765450936 | 3.036876478 |
| 91 | 3.216733282 | 8.750217519 | 0.721370568 |
| 92 | 2.19128707 | 6.244863307 | 0.671076885 |
| 93 | 1.73 | 4.1 | 0.31 |
| 94 | 2.25711066 | 5.976495607 | 0.597254995 |
| 95 | 1.1 | 3.34 | 0.28 |
| 96 | 1.150952412 | 3.587660034 | 0.394931577 |
| 97 | 4.073720252 | 10.74465148 | 1.511421548 |
| 98 | 1.601056466 | 4.281801046 | 0.237828746 |
| 99 | 3.892156139 | 12.79151737 | 1.090966398 |
| 100 | 1.907885411 | 5.265558027 | 0.30699662 |
| 101 | 2.773154052 | 7.467883618 | 0.501464809 |
| 102 | 1.516357159 | 12.08339211 | 6.645369673 |
| 103 | 0.705143394 | 5.246587439 | 2.801277286 |
| 104 | 3.223297099 | 7.977883612 | 0.601790027 |
| 105 | 0.83 | 7.14 | 3.84 |
| 106 | 3.316563015 | 9.954111165 | 1.45306527 |
| 107 | 1.303013152 | 3.974820607 | 0.358180175 |
| 108 | 2.369972886 | 6.654596716 | 0.794286243 |
| 109 | 0.220211376 | 2.303982189 | 1.136280944 |
| 110 | 0.495549552 | 6.558157519 | 3.577819989 |
| 111 | 0.926906654 | 6.90992779 | 3.141148388 |
| 112 | 3.731246651 | 9.956414716 | 1.226309594 |
| 113 | 0.53 | 5.55 | 2.79 |
| 114 | 3.150143409 | 7.902044521 | 0.48412972 |
| 115 | 3.005381295 | 8.298245489 | 1.023547679 |
| 116 | 1 | 7.14 | 3.48 |
| 117 | 0.772122807 | 10.30317125 | 5.739601852 |
| 118 | 0.455956799 | 4.221199384 | 2.18644952 |
| 119 | 2.37 | 6.16 | 0.52 |
| 120 | 0.827604689 | 6.565954596 | 3.00756585 |
| 121 | 3.043653096 | 8.385442423 | 0.464799445 |
| 122 | 1.729995468 | 12.16623819 | 6.093780873 |
| 123 | 4.147154431 | 10.83234867 | 1.440753426 |
| 124 | 1.847901076 | 5.579473607 | 0.504877844 |
| 125 | 2.896561155 | 7.55853435 | 0.838350057 |
| 126 | 0.563302154 | 5.97311125 | 3.155420122 |
| Max | 6.06 | 16.06 | 6.64 |
| Min | 0.22 | 2.30 | 0.237 |
| Average | 2.12 | 7.29 | 1.61 |
| CV | - | - | 87.88% |

**Table S2**: Substance content statistics for the prediction set

| Prediction set | Capsanthin | Total carotenoids | Zeaxanthin |
| --- | --- | --- | --- |
| 1 | 2.147073954 | 5.931608181 | 0.414806397 |
| 2 | 2.779707517 | 7.588607722 | 0.637431526 |
| 3 | 0.671630536 | 6.288586167 | 2.990031845 |
| 4 | 0.426357892 | 3.278613802 | 1.821193234 |
| 5 | 2.239291817 | 5.406504802 | 0.250601065 |
| 6 | 1.911847457 | 5.685698892 | 0.406703992 |
| 7 | 3.310461315 | 8.119821296 | 0.55733273 |
| 8 | 2.621511392 | 6.998161751 | 0.49545066 |
| 9 | 2.586196526 | 8.269552226 | 1.153003889 |
| 10 | 3.791123226 | 9.940209528 | 1.615651542 |
| 11 | 2.86 | 7.21 | 0.74 |
| 12 | 2.21369087 | 5.931844521 | 0.482329809 |
| 13 | 2.272820911 | 6.52665932 | 0.509096935 |
| 14 | 3.961808784 | 10.98787009 | 1.589720865 |
| 15 | 1.583130837 | 11.19757762 | 5.590363411 |
| 16 | 1.456930513 | 4.469218139 | 0.348336977 |
| 17 | 3.055228044 | 8.368963514 | 0.707525583 |
| 18 | 2.342961426 | 6.591262994 | 0.500286739 |
| 19 | 2.14639779 | 6.74294407 | 1.08557215 |
| 20 | 2.463164917 | 5.974118313 | 0.391597021 |
| 21 | 1.22 | 11.39 | 6.22 |
| 22 | 2.150732289 | 7.204537044 | 1.467510036 |
| 23 | 0.90269118 | 6.122372159 | 2.828944806 |
| 24 | 0.6 | 6.97 | 3.42 |
| 25 | 2.82 | 7.69 | 0.8 |
| 26 | 0.352884292 | 5.39170414 | 3.140927589 |
| 27 | 1.956894092 | 5.993688552 | 0.736831223 |
| 28 | 1.157428417 | 10.44801544 | 5.129142342 |
| 29 | 2.784178968 | 6.964409454 | 0.71614297 |
| 30 | 0.52 | 4.17 | 1.95 |
| 31 | 0.645829935 | 5.157547518 | 2.866603113 |
| 32 | 0.49 | 5.02 | 2.38 |
| 33 | 1.852898857 | 5.392235167 | 0.72097655 |
| Max | 3.96 | 11.39 | 6.22 |
| Min | 0.35 | 3.27 | 0.25 |
| Average | 1.94 | 6.95 | 1.65 |
| CV | - | - | 95.84% |

**Table S3**: Consistency test of spectral feature distribution in the calibration/prediction set

| Bands | KS-statistic--D | p |
| --- | --- | --- |
| 365 nm | 0.161 | 0.47 |
| 405 nm | 0.146 | 0.587 |
| 430 nm | 0.122 | 0.789 |
| 450 nm | 0.106 | 0.901 |
| 470 nm | 0.104 | 0.914 |
| 490 nm | 0.096 | 0.952 |
| 515 nm | 0.148 | 0.568 |
| 540 nm | 0.157 | 0.502 |
| 570 nm | 0.189 | 0.279 |
| 590 nm | 0.239 | 0.09 |
| 630 nm | 0.192 | 0.267 |
| 645 nm | 0.143 | 0.618 |
| 660 nm | 0.122 | 0.791 |
| 690 nm | 0.114 | 0.851 |
| 780 nm | 0.131 | 0.721 |
| 850 nm | 0.11 | 0.879 |
| 880 nm | 0.135 | 0.684 |
| 940 nm | 0.159 | 0.486 |
| 970 nm | 0.189 | 0.279 |
| Max | 0.239 | 0.952 |
| Min | 0.096 | 0.09 |

**Table S4**: Ablation experiment performance comparison

| **Model** | **Input Features** | **Rp²** | **RMSE_p_​** | **Improvement vs. M-Spectral** |
| --- | --- | --- | --- | --- |
| M-Spectral | Spectral only | 0.7639 | 0.7102 | - |
| M-Chemical | Chemical only | 0.4425 | 1.221 | - |
| M-S+R (Random) | Spectral + random noise | 0.5750 | 1.0393 | - |
| M-S+N (Noisy) | Spectral + perturbed chemical data | 0.8253 | 0.5174 | +8.04% |
| M-MCSF​ | Spectral + authentic chemical data​ | 0.9318 | 0.4172​ | +21.98% |

**Table S5**. Comparison of model complexity and inference time across different spectral bands

| **Spectral band types** | **Model** | **Parameters** | **Inference Time (ms)** | ***Rp*²​** |
| --- | --- | --- | --- | --- |
| All spectral bands | 1D-CNN | 2177 | 0.288 | 0.7639 |
|  | MCSF | 3929 | 0.364 | 0.9318 |
| Characteristic spectral band | 1D-CNN | 2033 | 0.274 | 0.6730 |
|  | MCSF | 1969 | 0.273 | 0.9211 |


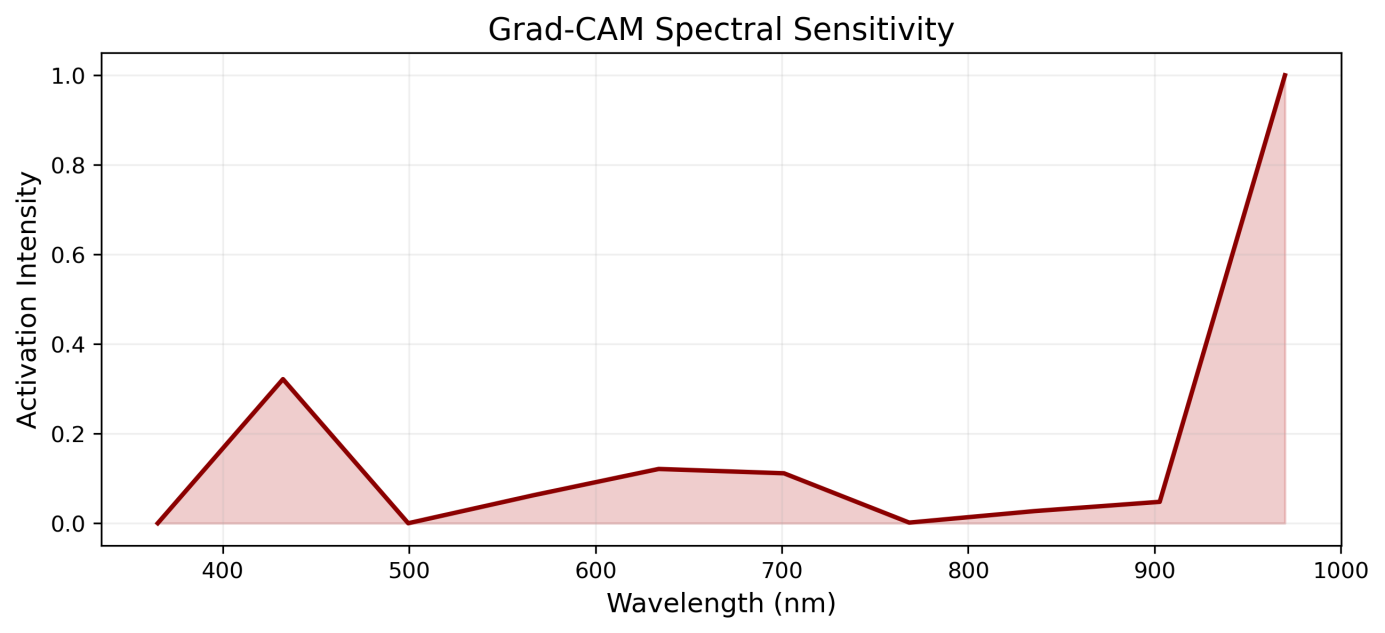


**Fig. S1**: Grad-CAM heat map of spectral activation intensities of the MCSF model in different bands


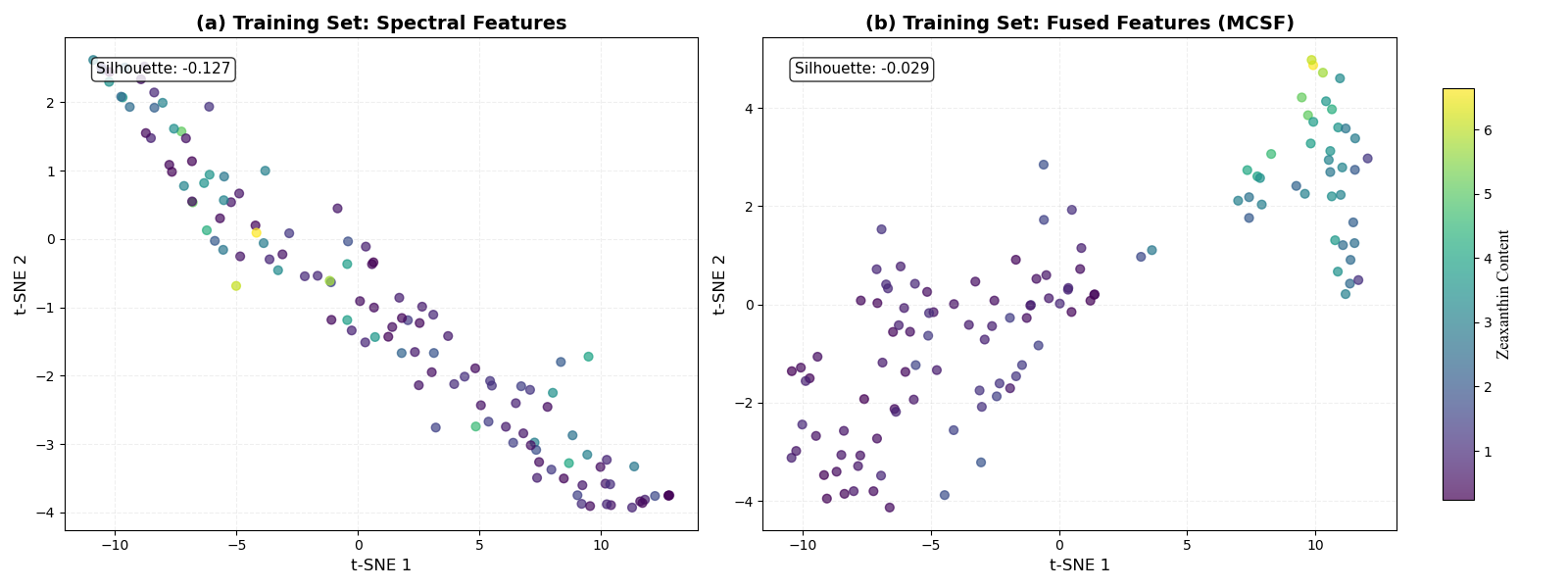
**Fig. S2.** t-SNE visualization of feature space from the spectral-only model and the multimodal fusion model. Results of the (a) M-Spectral model and (b) MCSF model.


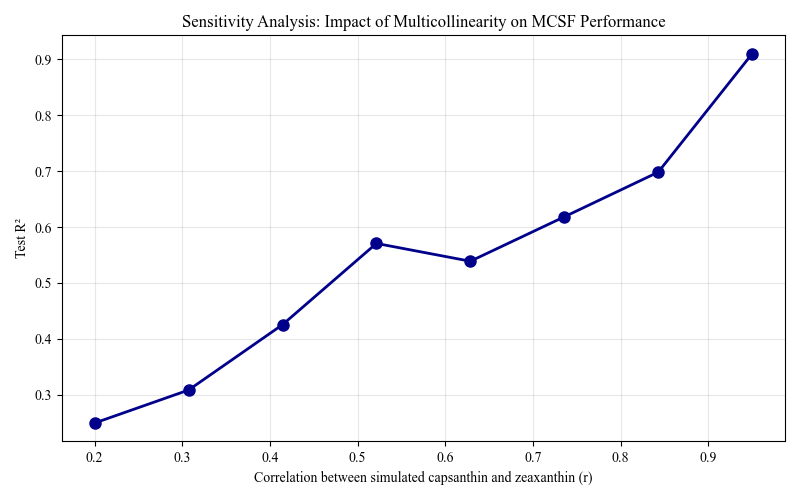


**Fig. S3.**Sensitivity analysis of the MCSF model under varying correlation between simulated capsanthin and true zeaxanthin content.


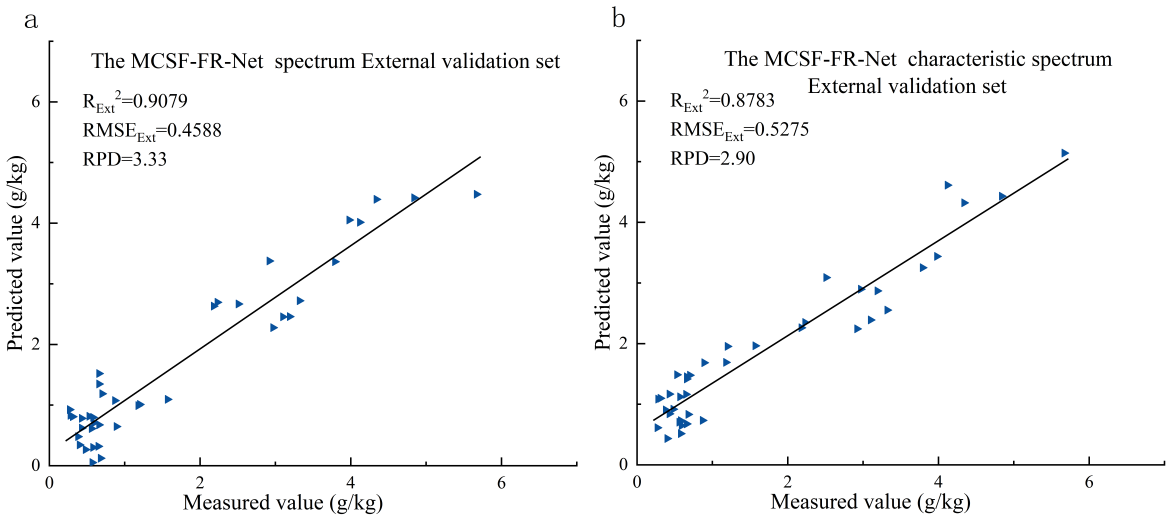


**Fig. S4.** External validation performance of the full-spectrum and characteristic-spectrum models. (a) the full-spectrum (b) the characteristic-spectrum.
